# Low-Concentration Ammonia Gas Sensors Manufactured Using the CMOS–MEMS Technique

**DOI:** 10.3390/mi11010092

**Published:** 2020-01-15

**Authors:** Wei-Chun Shen, Po-Jen Shih, Yao-Chuan Tsai, Cheng-Chih Hsu, Ching-Liang Dai

**Affiliations:** 1 Department of Mechanical Engineering, National Chung Hsing University, Taichung 402, Taiwan; deer5300158@hotmail.com; 2 Department of Biomedical Engineering, National Taiwan University, Taipei 106, Taiwan; pjshih@ntu.edu.tw; 3 Department of Bio-Industrial Mechatronics Engineering, National Chung Hsing University, Taichung 402, Taiwan; yctsaii@dragon.nchu.edu.tw; 4 Department of Electro-Optical Engineering, National United University, Miaoli 360, Taiwan; cchsu920624@nuu.edu.tw

**Keywords:** ammonia, gas sensor, low concentration, CMOS process, MEMS

## Abstract

This study describes the fabrication of an ammonia gas sensor (AGS) using a complementary metal oxide semiconductor (CMOS)–microelectromechanical system (MEMS) technique. The structure of the AGS features interdigitated electrodes (IDEs) and a sensing material on a silicon substrate. The IDEs are the stacked aluminum layers that are made using the CMOS process. The sensing material; polypyrrole/reduced graphene oxide (PPy/RGO), is synthesized using the oxidation–reduction method; and the material is characterized using an electron spectroscope for chemical analysis (ESCA), a scanning electron microscope (SEM), and high-resolution X-ray diffraction (XRD). After the CMOS process; the AGS needs post-processing to etch an oxide layer and to deposit the sensing material. The resistance of the AGS changes when it is exposed to ammonia. A non-inverting amplifier circuit converts the resistance of the AGS into a voltage signal. The AGS operates at room temperature. Experiments show that the AGS response is 4.5% at a concentration of 1 ppm NH_3_; and it exhibits good repeatability. The lowest concentration that the AGS can detect is 0.1 ppm NH_3_

## 1. Introduction

Gas sensors are used to detect harmful gases and avoid the danger of harmful gases being inhaled. Many chronic diseases are due to modern society’s complex lifestyle and aging population [1]. Many studies about human diseases discuss possible means of monitoring health by analyzing the breath. This diagnostic method is non-invasive, portable, and rapid. Some volatile organic compounds in the human breath occur in abnormally large quantities if the body is not in good health [1]. The concentration of NH_3_ in the breath is only 0.2 to 0.5 ppm in healthy subjects [2]. The breath of patients with kidney problems demonstrates a NH_3_ concentration that is significantly higher than that for healthy subjects (mean: 5 ppm) [3]. When the function of the kidneys decreases, nitrogenous waste accumulates in the human body. Nitrogenous waste is released via the respiratory system, so NH_3_ concentration in the patients’ breath is higher than that of healthy subjects.

Gas sensors that are used to detect hydrogen sulfide [4], carbon dioxide [5], hydrogen [6], nitrogen dioxide [7], and formaldehyde [8] have been miniaturized using microelectromechanical system (MEMS) technology. Gas microsensors that use this technology are quiet and highly sensitive, feature low power consumption, and can be mass-produced easily. Many studies use MEMS technology to fabricate ammonia sensors. Table 1 lists the process method, sensing material, and working temperature of various ammonia gas sensors. Peng [9] used the MEMS process to produce an ammonia gas sensor (AGS) with a micro-hotplate that consumed less power. The AGS used nanoparticulate tin dioxide as the gas sensing material, which was prepared using the hydrothermal method. The sensing material was coated on the micro-hotplate. The working temperature (WT) of the AGS was 225 °C, and the AGS response was 23.94 at a concentration of 120 ppm ammonia. Kim [10] presented an AGS that was fabricated using silicon micromachining. The AGS structure contained a sensing material and a suspended micro-heater. The sensing material was a graphene sheet that was deposited on the suspended micro-heater. The micro-heater gave a WT that was appropriate for the sensing material, and the power consumption of the heater was 1 mW. The AGS had a response of 0.049 at 18.8 sccm ammonia for 70 s. Prajesh [11] used a microfabrication process to make an AGS, which consisted of a sensing film, a micro-heater, and interdigitated electrodes (IDEs). The micro-heater and the IDEs were constructed on an oxide membrane, and the sensing material was deposited onto the IDEs. The power consumption of the micro-heater was 98 mW and the WT for the AGS was 230 °C. The AGS response was 40% at a concentration of 4 ppm NH_3_. Lee [12] proposed an AGS that was manufactured using MEMS technology. The sensing film for the AGS consisted of tungsten trioxide powders with a Ru sol mixture that was synthesized using the sol–gel process. This was coated onto a MEMS membrane with electrodes and a heater. The heater gave a WT of 333 °C for the sensing film. The AGS had a response of 0.34 at a concentration of 5 ppm NH_3_. Wu [13] used micromachining and an inkjet printing process to produce an ammonia sensor to analyze breath. The AGS contained a micro-hotplate and a sensing material. The sensing material was graphene metal oxide, and this was deposited on the micro-hotplate using an inkjet printing process. The WT for the sensing film was achieved using the micro-hotplate. The response of the AGS was 15 at a concentration of 10 ppm NH_3_. Patois [14] produced an AGS using a silicon microfabrication process. The AGS worked at room temperature. The structure of the AGS featured an array of microelectrodes and a sensing film. The microelectrode array was produced using a silicon microfabrication process. The sensing film was polypyrrole, and this was deposited on the microelectrode array by electrochemical deposition. The AGS had a response of 16% at a concentration of 40 ppm NH_3_, and the lowest concentration detected by the AGS was 3 ppm. Tiwari [15] produced an AGS using a micromachining process. The AGS was made on a glass substrate with IDEs, and a sensing film was deposited onto the IDEs. The sensing material was pyrrole/reduced graphene oxide. The AGS operated at room temperature. The lowest concentration that was detected by the AGS was 3 ppm. The response of the AGS was 1.1% at a concentration of 3 ppm. The lowest concentration to be detected by any of the AGSs [9,10,11,12,13,14] was higher than 1 ppm. This study produces an AGS using the complementary metal oxide semiconductor–microelectromechanical system (CMOS–MEMS) technique. The lowest concentration of ammonia gas that can be detected by this AGS is 0.1 ppm. It has the potential to be used for breath analysis.

CMOS–MEMS was used to produce micro-actuators [16,17] and microsensors [18,19,20]. This process allowed the integration of micro-devices and integrated circuits on a chip, which reduced noise and enhanced the micro-devices’ performance. This study details the production of an AGS using CMOS–MEMS. This AGS is a resistive type. When the AGS detects ammonia gas, its resistance changes. A non-inverting amplifier circuit converts the change in the resistance of the AGS to a voltage output. The AGS contains a sensing material and IDEs. The sensing material is polypyrrole/reduced graphene oxide (PPy/RGO), which was synthesized using the oxidation–reduction method.

## 2. Structure of the Ammonia Sensor

**Figure 1:**
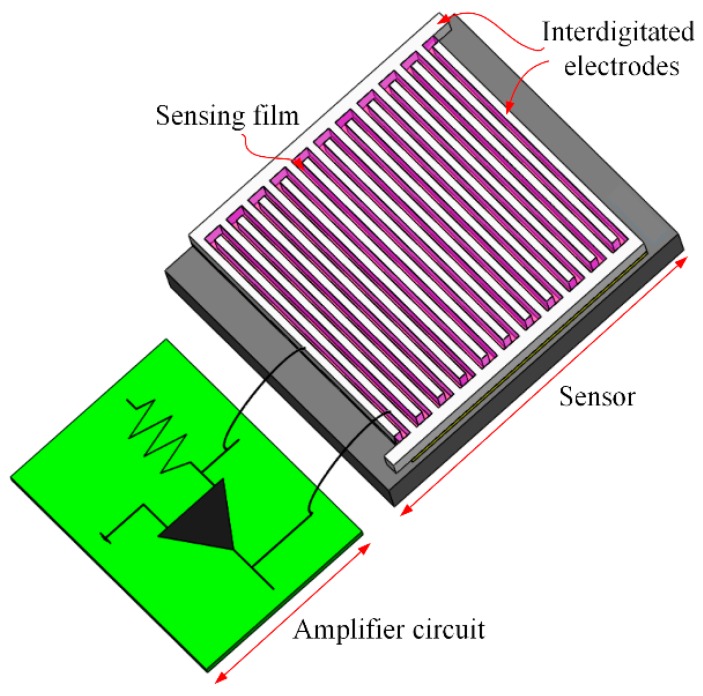
Ammonia gas sensor structure.

Figure 1 shows the AGS structure. The CMOS process was used to produce the AGS. As shown in Figure 1, the AGS consists of IDEs and a sensing film on a silicon substrate. The area of the AGS is 1.2 mm^2^. The IDEs are formed by stacking aluminum layers in the CMOS process. Each finger of the IDEs has a length of 700 μm, a width of 20 μm, and a thickness of 7 μm. The gap between the IDEs is 10 μm. The sensing film is coated onto the IDEs. The oxidation–reduction method is used to synthesize the sensing film, which consists of PPy/RGO. Most gas sensors [6,7,8,9,10,11,12] have a heater to generate the WT for the sensitive film, but this study produced an AGS without a micro-heater. This AGS operates at room temperature, so it is more efficient in terms of power consumption.

The RGO in the sensing film increases the conductivity of the film. The PPy in the sensing film provides the main reaction for ammonia. The mechanism for the reaction between the PPy/RGO film and ammonia is described as follows [21]:

(1) $$PPy^{+}A^{-}+NH_{3}⇌PPy{\left(-H\right)}^{0}+NH_{4}^{+}A^{-}$$

When the NH_3_ molecules make contact with the PPy, the doublet of nitrogen in NH_3_ loses an electron to the nitrogen of the polymer backbone, which results in the formation of an ammonium ion ${\mathrm{NH}}_{4}^{+}$, which is similar to a deprotonation process [21]. An electron is transferred from NH_3_ to the PPy/RGO, and the density of positive holes in PPy/RGO decreases, so the resistance of PPy/RGO increases. Therefore, as the sensing film comes into contact with ammonia, the resistance of the AGS changes.

**Figure 2:**
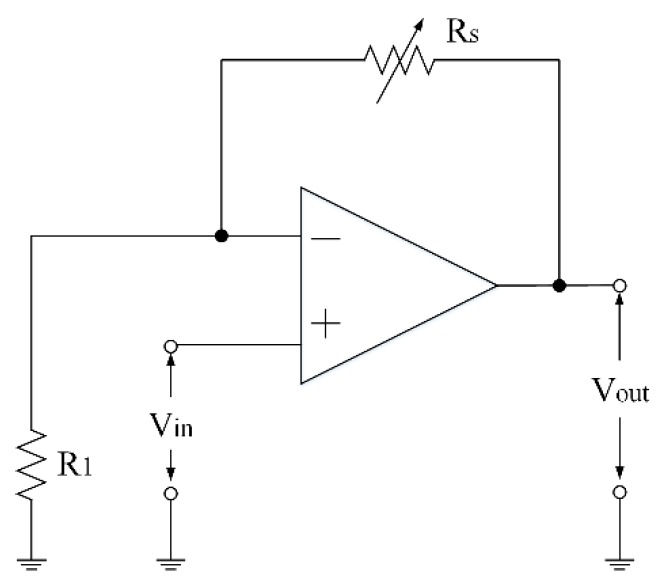
Non-inverting amplifier circuit for the ammonia gas sensor (AGS).

**Figure 3:**
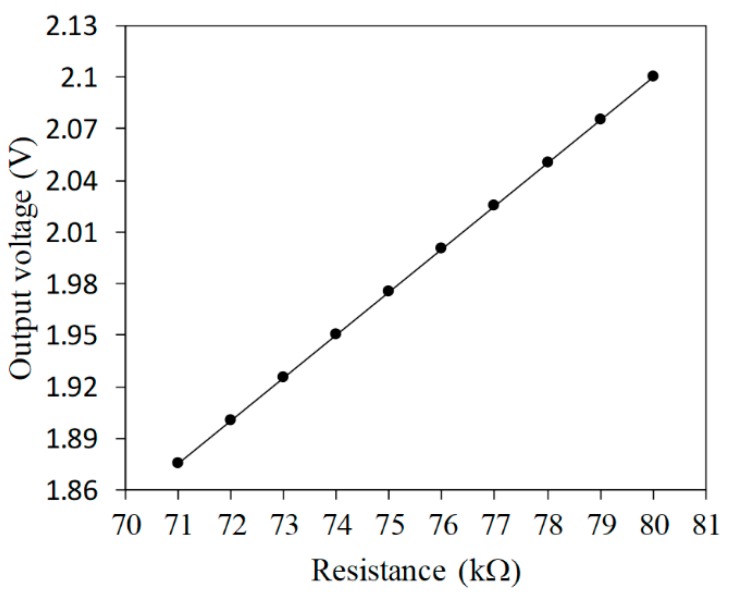
Evaluated output voltage for the AGS.

As shown in Figure 1, a non-inverting amplifier circuit is joined to the AGS using a wire bonding process. This circuit converts the resistance of the AGS into a voltage output. Figure 2 shows the non-inverting amplifier circuit for the AGS. The output voltage for the non-inverting amplifier circuit is expressed as follows [22]:

(2) $$V_{out}=V_{in}\times \left(1+\frac{R_{s}}{R_{1}}\right),$$

where $V_{out}$ represents the output voltage, $V_{in}$ is the input voltage, $R_{s}$ is the resistance of the AGS, and $R_{1}$ is the resistance of the circuit. The output voltage of the circuit is proportional to the resistance of the AGS. Equation (2) is used to calculate the output voltage for the circuit. Figure 3 displays the evaluated output voltage of the circuit for the AGS. The resistance of $R_{1}\;$ is 4 kΩ and the input voltage $V_{in}$ is 0.1 V. The resistance of the AGS varies from 71 to 80 kΩ. As shown in Figure 3, the evaluated results show that the output voltage for the circuit increases from 1.875 to 2.1 V when the resistance increases from 71 to 80 kΩ.

## 3. Preparation of the Sensing Film

PPy/RGO was synthesized using the oxidation–reduction method. The steps of the PPy/RGO preparation are described as follows [23]: 2 g of flaked graphite powder, 0.5 g of sodium nitrate, and 25 mL of sulfuric acid were stirred vigorously for 30 min, followed by cooling at 4 °C for 30 min. A 4 g quantity of potassium permanganate was added into the solution, which was then vigorously stirred at 40 °C for 60 min. A 50 mL amount of deionized water was added into the solution, which was vigorously stirred at 98 °C for 5 min. Hydrogen peroxide was added into the solution, and stirred until the solution was mixed uniformly. The result, which was graphite oxide (GO), was filtered and washed with hydrochloric acid and deionized water. The product (GO) was dried at 40 °C for 12 h. Pyrrole (5 mL), ethanol (10 mL), GO (10 mg), and deionized water were mixed by vigorous stirring at 80 °C for 4 h. The resultant product was filtered and washed with dimethylformamide and deionized water. The product (PPy/RGO) was then deposited on the substrate and baked at 90 °C for 1 h.

**Figure 4:**
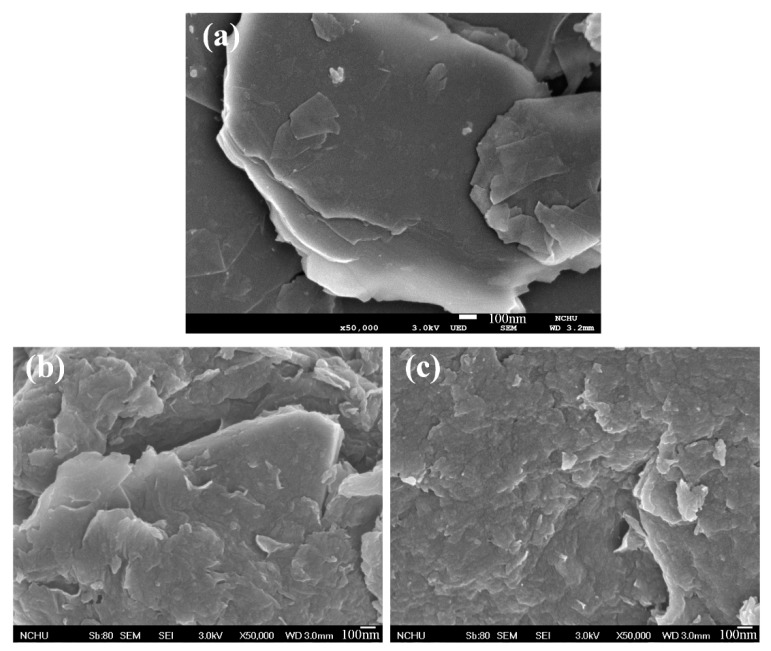
Scanning Electron Microscope (SEM) images; (**a**) graphite, (**b**) graphene oxide (GO), and (**c**) polypyrrole/reduced graphene oxide (PPy/RGO).

**Figure 5:**
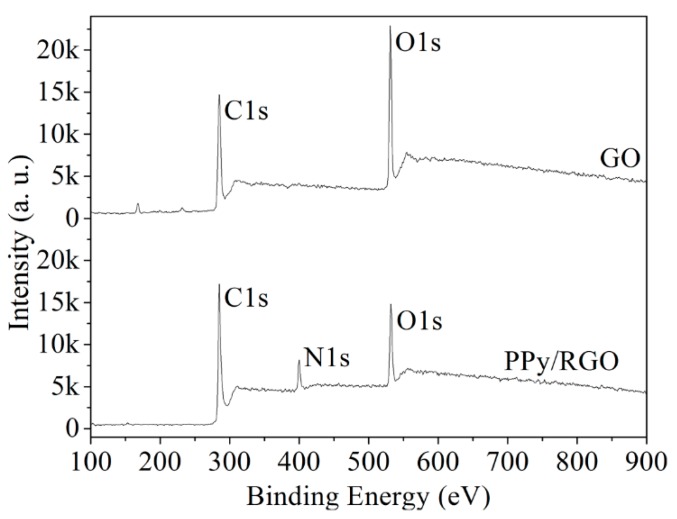
Full electron spectroscope for chemical analysis (ESCA) energy spectrum for GO and PPy/RGO.

**Figure 6:**
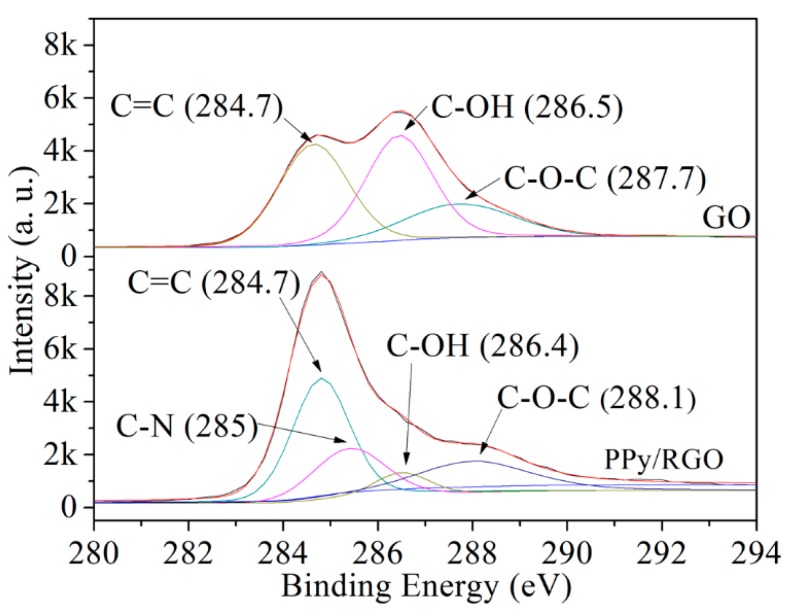
De-convoluted ESCA energy spectrum for GO and PPy/RGO.

A SEM, an ESCA, and an XRD were used to measure the characteristics of graphite, GO, and PPy/RGO film. Figure 4 shows the SEM images for graphite, GO, and PPy/RGO. Figure 4a shows that the graphite has a flat, flaky structure. Figure 4b shows that the GO has a rippled and folded structure due to the presence of oxygen functional groups. Figure 4c shows that the PPy/RGO has a corrugated and uniform convex structure, because the surface of RGO is capped by the oxidation product of pyrrole. Figure 5 shows the full ESCA energy spectrum for GO and PPy/RGO. The peak (N1s) at 400 eV is attributed to the presence of nitrogen from the adsorbed polypyrrole on the RGO [24]. Figure 6 shows the de-convoluted ESCA energy spectrum for GO and PPy/RGO. The peak at 285 eV (C–N) is attributed to nitrogen in the PPy/RGO and non-covalent bonding between the polypyrrole and RGO [24]. 

**Figure 7:**
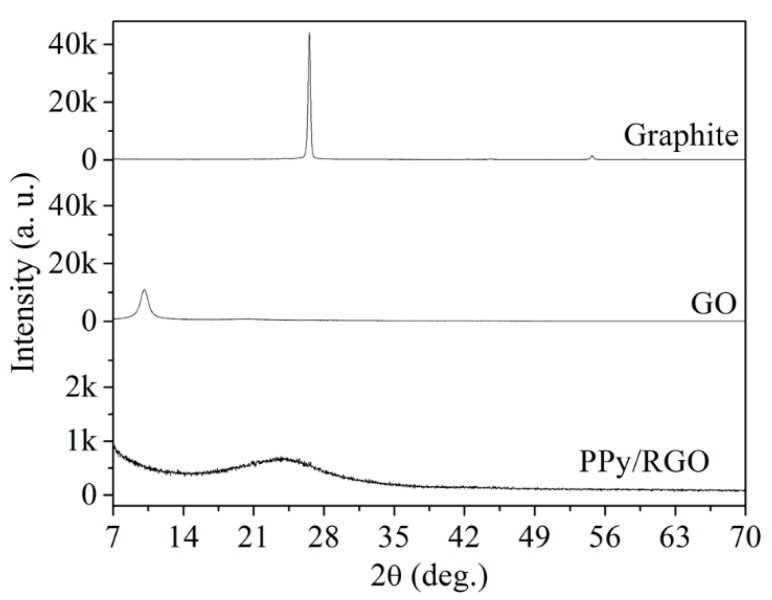
X-ray diffraction (XRD) patterns for graphite, GO, and PPy/RGO.

Figure 7 shows the XRD patterns for graphite, GO, and PPy/RGO. The graphite spectrum features a strong diffraction peak at 26.5° because there is a uniform and tightly layered structure. The diffraction peak for the GO is shifted to 10.1°, and the peak intensity shows a decrease, but the diffraction peak for GO at 10.1° is a factor of the expanded layer structure, because of the presence of oxygen functional groups on the GO surface. The PPy/RGO pattern features a peak at 24° that is small and wide because of the amorphous structure of PPy polymer and the covering of RGO by polypyrrole. 

## 4. Fabrication of the Ammonia Sensor

**Figure 8:**
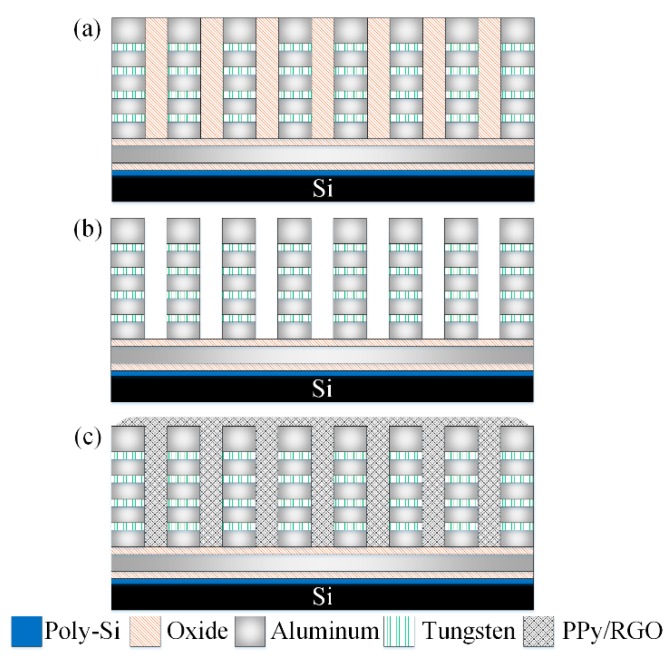
Manufacturing flow for the AGS; (**a**) after the CMOS process, (**b**) etching the oxide layer, and (**c**) coating the sensitive film.

**Figure 9:**
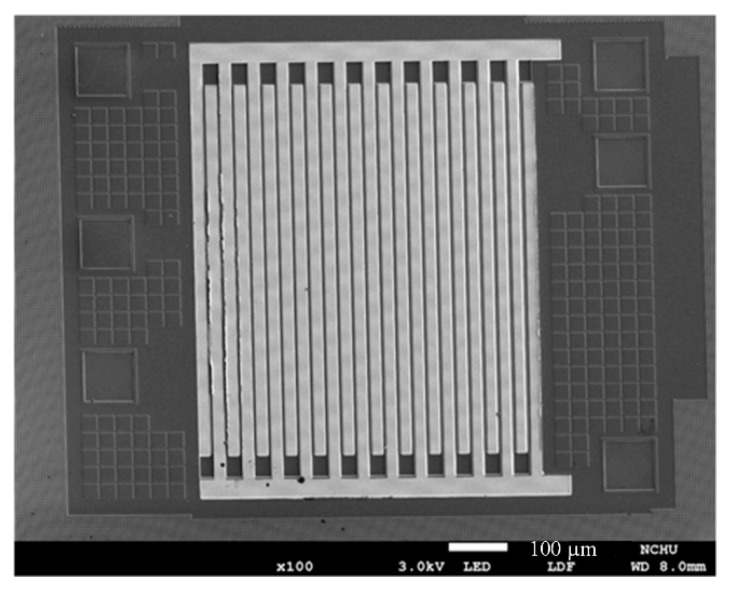
SEM image of the AGS after etching the oxide layer.

**Figure 10:**
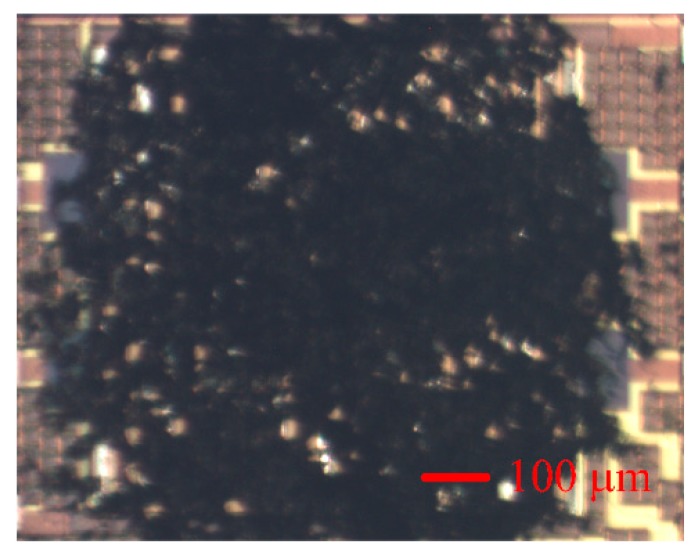
Optical image of the AGS after coating the sensing film.

The AGS was produced using the CMOS process of the Taiwan Semiconductor Manufacturing Company [25]. Figure 8 shows the manufacturing flow of the AGS. Figure 8a shows the cross-sectional view of the AGS after the CMOS process. After that process, the AGS needed post-processing to etch an oxide layer and to deposit the sensing film. The oxide layer, located between the IDEs, needed to be etched. Figure 8b demonstrates the etching of the oxide layer. A wet etching process using a buffered oxide etch (BOE) was employed to remove the oxide layer and to produce the IDEs [26]. The IDEs have a stacked structure that comprises aluminum and tungsten metal. The etching rate of the oxide was about 250 nm/min. The wet etching needed to be timed, to avoid over-etching. The etching time was about 28 min. Figure 8c shows the coating for the sensing films. The sensing film was coated onto the IDEs using a microdropper. Figure 9 displays an optical image of the AGS after the oxide layer is etched. Figure 10 shows a SEM image of the AGS after the sensing film is coated.

## 5. Results and Discussion

The characteristics of the AGS were measured in a test chamber, using an LCR meter and a digital multimeter. The LCR meter was used to measure the resistance of the AGS. The digital multimeter was utilized to detect the output voltage of the amplifier circuit. The test chamber contained a control valve, a flow rate sensor, and a pump. The control value controlled the concentration of ammonia in the test chamber, and the flow rate sensor monitored the concentration of ammonia in the test chamber. The pump drained the ammonia from the test chamber after testing. The AGS was located in the test chamber.

**Figure 11:**
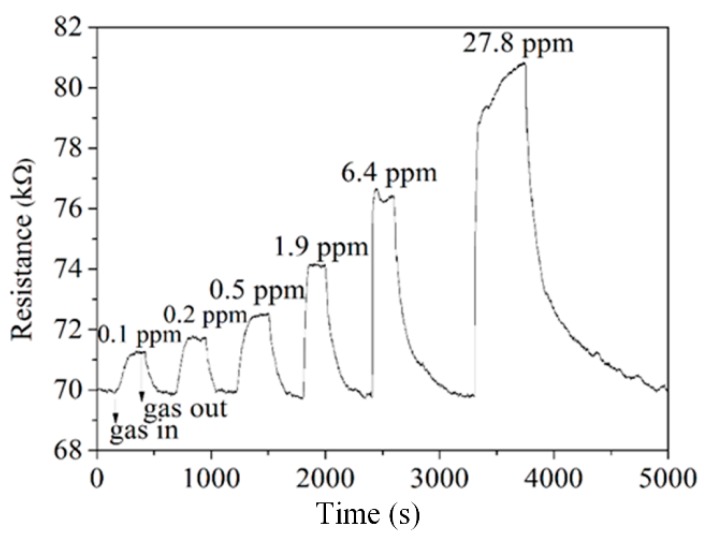
Change in the resistance of the AGS for different concentrations of ammonia.

**Figure 12:**
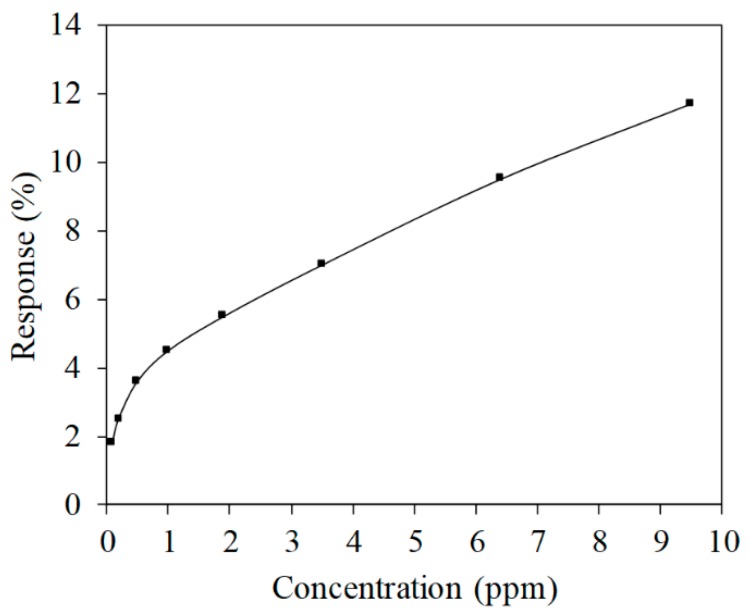
Response of the AGS.

The difference in the resistance of the AGS was measured. The LCR meter measured the change in the resistance of the AGS for different concentrations of ammonia. Figure 11 shows the behavior of the AGS for different concentrations of ammonia. The resistance of the AGS was 70 kΩ in air. When the concentration of ammonia in the test chamber was 0.1 ppm, the resistance of the AGS changed to 71.2 kΩ. This result showed that the AGS could detect 0.1 ppm ammonia. The AGS had a response time of 118 s at 0.1 ppm ammonia, and a recovery time of 122 s at 0.1 ppm ammonia. As shown in Figure 11, the resistance of the AGS is 74.3 kΩ at an ammonia concentration of 1.9 ppm. A gas sensor’s response is an important performance metric. The response of the AGS is defined as follows:

(3) $$S=\frac{R_{g}-R_{a}}{R_{a}}\times 100\%,$$

where *R_a_* represents the resistance of the AGS in air and *R_g_* is the change in the resistance of the AGS in ammonia. Figure 12 shows the response of the ammonia sensor. The response of the AGS increased from 1.83% to 11.7% when the ammonia concentration changed from 0.1 to 9.5 ppm.

**Figure 13:**
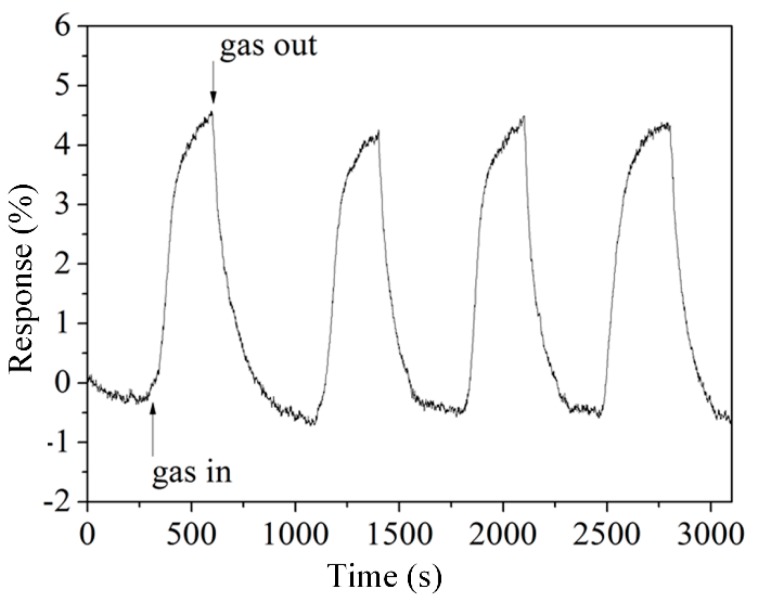
Cycle test for the AGS at 1 ppm ammonia.

To determine the repeatability of the AGS, the AGS was tested using a cycle test. Figure 13 shows the results of the cycle test for the AGS at 1 ppm ammonia. The response of the AGS at 1 ppm ammonia was about 4.5% for each cycle test. These results show that the AGS demonstrated good repeatability.

**Figure 14:**
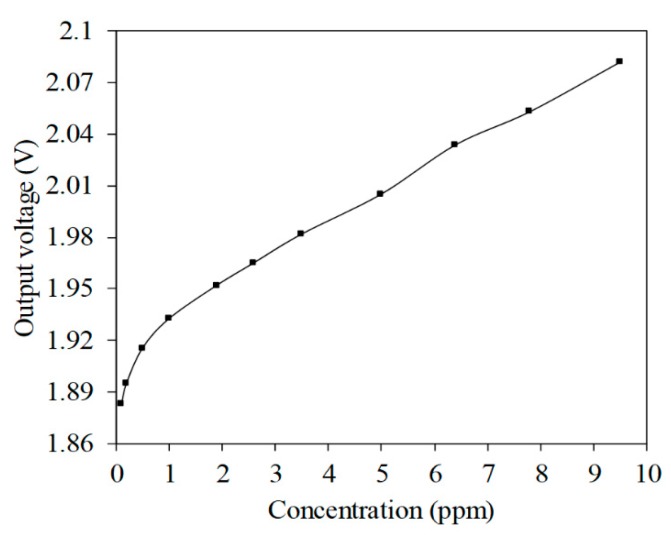
Output voltage of the AGS.

The AGS was tested with a non-inverting amplifier circuit. A power supply generated a bias voltage to the amplifier circuit and a digital multimeter measured the output voltage from the amplifier circuit. Figure 14 shows the output voltage from the AGS with an amplifier circuit. The AGS had an output voltage of 1.883 V at 0.1 ppm NH_3_. The output voltage from the AGS was 1.932 V at 1 ppm NH_3_, and 2.088 V at 9.5 ppm NH_3_. The output voltage for the AGS changed by 153 mV when the concentration of ammonia increased from 1 to 9.5 ppm.

Table 2 lists the performance for various ammonia gas sensors, where *R_a_* is the resistance of the AGSs in air, and *R_g_* is the change in the resistance of the AGSs in ammonia. The lowest concentration detectability for the AGSs produced by Lee [12] and Wu [13] is 1 ppm. The lowest concentration detectability for the AGS fabricated by Khuspe [27] is 10 ppm. The lowest concentration detectability for the AGS produced by this work is 0.1 ppm, which is a better figure than that for the AGSs produced by Lee [12], Wu [13], Patois [14], Tiwari [15], Bandgar [28], and Khuspe [27].

**Figure 15:**
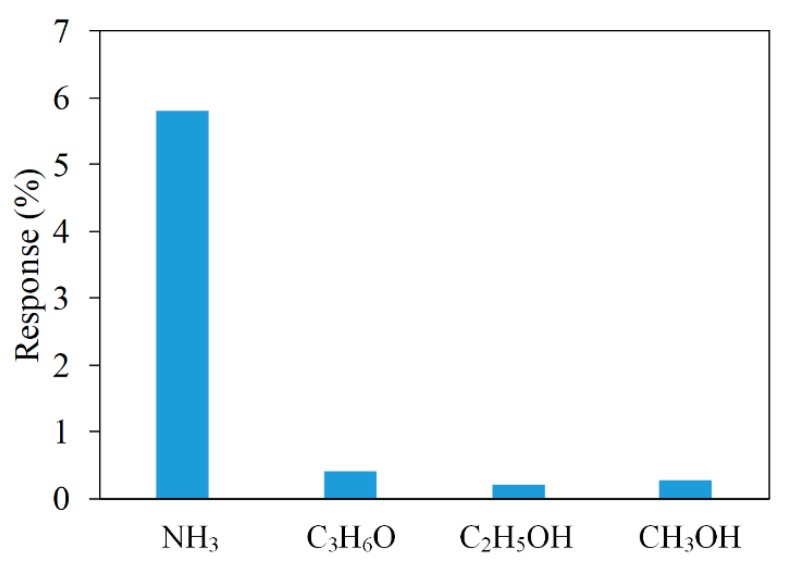
Response of the AGS under different gases.

The AGS was measured with different gases to figure out its selectivity. Figure 15 depicts the response of the AGS under ammonia, acetone, ethanol, and methanol. In this test, the sensor operated at room temperature and the concentration of all gases was controlled at 2 ppm. The tested results showed that the AGS had the best response to ammonia, with 5.8% at 2 ppm. Therefore, the AGS had exceptional selectivity for sensing ammonia.

## 6. Conclusions

A gas sensor for measuring low concentrations of ammonia gas was manufactured using the CMOS–MEMS technique. The AGS needed post-processing after the CMOS process. The post-processing involved wet etching of the BOE to etch the oxide layer, and the sensing film was then deposited onto the IDEs. The sensing film was PPy/RGO, which was synthesized using the oxidation–reduction method. Most ammonia gas sensors use a micro-heater [9,10,11,12,13] to generate an appropriate WT of 225–330 °C for the sensing film, so the power consumption is quite high. This study details the production of an AGS without a micro-heater, which operates at room temperature, so it is very efficient in terms of power consumption. The lowest concentration that can be detected by the AGS is 0.1 ppm NH_3_, and the AGS features good repeatability, so it can be used for breath analysis. The experimental results showed that the AGS’s response was 4.5% at a concentration of 1 ppm NH_3_. The resistance of the AGS was converted into an output voltage using a non-inverting amplifier circuit. The results showed that the output voltage of the AGS with an amplifier circuit changed by 153 mV when the concentration of ammonia increased from 1 to 9.5 ppm.

## Figures and Tables

**Table 1:** Process and characteristic of various ammonia gas sensors (AGSs).

Authors	Process	Sensing Material	Heater	Working Temperature
Peng [9]	MEMS (microelectromechanical system)	Tin dioxide	Yes	225 °C
Kim [10]	Micromachining	Graphene sheet	Yes	200 °C
Prajesh [11]	Microfabrication	Metal oxide	Yes	230 °C
Lee [12]	MEMS	Tungsten trioxide/Ru	Yes	333 °C
Wu [13]	Micromachining/inkjet printing	Graphene metal oxide	Yes	325 °C
Patois [14]	Microfabrication	Polypyrrole	No	Room temperature
Tiwari [15]	Micromachining	Pyrrole/reduced graphene oxide	No	Room temperature

**Table 2:** Performances of various ammonia gas sensors.

Authors	Gas	Response	Detectability	Formula of Response
Lee [12]	NH_3_	3.4 (5 ppm)	1 ppm	*S = R_g_/R_a_*
Wu [13]	NH_3_	15 (10 ppm)	1 ppm	*S = R_g_/R_a_*
Patois [14]	NH_3_	16% (40 ppm)	3 ppm	*S = (R_g_− R_a_)/R_a_* × 100%
Tiwari [15]	NH_3_	1.1% (3 ppm)	3 ppm	*S = (R_g_− R_a_)/R_a_* × 100%
Bandgar [28]	NH_3_	72% (100 ppm)	2.5 ppm	*S = (R_g_− R_a_)/R_a_* × 100%
Khuspe [27]	NH_3_	91% (100 ppm)	10 ppm	*S = (R_g_− R_a_)/R_a_* × 100%
Merian [29]	NH_3_	350% (5 ppm)	0.1 ppm	*S = (R_g_− R_a_)/R_a_* × 100%
This work	NH_3_	4.5% (1 ppm)	0.1 ppm	*S = (R_g_− R_a_)/R_a_* × 100%
